# Hyperoxia: Effective Mechanism of Hyperbaric Treatment at Mild-Pressure

**DOI:** 10.3390/ijms25020777

**Published:** 2024-01-08

**Authors:** Mariana Cannellotto, Ali Yasells García, María Silvina Landa

**Affiliations:** 1 Research Department, International Hyperbaric Medicine and Research Association (IHMERA), Buenos Aires 1429, Argentina; 2 UniVida Medical Centers, Miami, FL 33126, USA

**Keywords:** hyperbaric oxygen treatment (HBOT), hyperoxia, mild pressure, PO_2_, reactive oxygen species (ROS)

## Abstract

HBOT increases the proportion of dissolved oxygen in the blood, generating hyperoxia. This increased oxygen diffuses into the mitochondria, which consume the majority of inhaled oxygen and constitute the epicenter of HBOT effects. In this way, the oxygen entering the mitochondria can reverse tissue hypoxia, activating the electron transport chain to generate energy. Furthermore, intermittent HBOT is sensed by the cell as relative hypoxia, inducing cellular responses such as the activation of the HIF-1α pathway, which in turn, activates numerous cellular processes, including angiogenesis and inflammation, among others. These effects are harnessed for the treatment of various pathologies. This review summarizes the evidence indicating that the use of medium-pressure HBOT generates hyperoxia and activates cellular pathways capable of producing the mentioned effects. The possibility of using medium-pressure HBOT as a direct or adjunctive treatment in different pathologies may yield benefits, potentially leading to transformative therapeutic advancements in the future.

## 1. Introduction

Oxygen is the essential component of cellular aerobic metabolism. It enters the respiratory pathways and blood vessels through convection, then diffuses through the capillary wall to the interstitium, with its ultimate destination being the mitochondria, where it plays a role in cellular respiration.

Oxygen is utilized as a therapeutic agent, not only for treating patients in need of oxygen supplementation, but it is also considered a drug capable of inducing targeted clinical responses, such as hyperbaric oxygen therapy (HBOT) [1] or therapies utilizing the “Normobaric Oxygen Paradox” or the “Hyperoxia-Hypoxia Paradox” [2,3]. These effects may vary in response to different pressures used, oxygen exposure durations, and the number of sessions for various pathologies. Therefore, it is necessary to standardize these parameters and characterize the responses based on the patient’s specific needs.

The aim of this review is to demonstrate that hyperbaric treatment using 1.5 atm generates an oxygen increase in blood and tissues, impacting cellular metabolism and producing beneficial effects for the therapy of various pathologies.

## 2. Hyperoxia

Hyperoxia is defined as an increase in the presence of tissue and plasma oxygen at concentrations higher than normal. The term “hyperoxia” is widely used in scientific language, and the word “HYPEROXIA” is documented in 23,957 articles on Pubmed Central (PMC)-nih.gov and in 9541 publications on PubMed-nih.gov. Given its clinical relevance, this concept is also extensively employed in the 243 clinical studies reported on ClinicalTrials.gov.

Hyperoxia can be achieved in situations where individuals are provided with oxygen supplementation (oxygen therapy) under normobaric or hyperbaric conditions. Normobaric oxygen supplementation, delivered through a mask, is used as a therapy for various medical conditions. Hyperbaric oxygen therapy (HBOT) involves breathing high concentrations of oxygen (O_2_) (~100%) within a pressurized chamber set above normal atmospheric pressure (at sea level, 1.0 absolute atmospheres or atm). For clinical usage, the pressure must be at least 1.4 atm according to the UHMS (Undersea and Hyperbaric Medical Society) guidelines, as stated in the 14th Edition 2019-Hyperbaric Oxygen Therapy Indications. Hyperoxia is the causal factor behind the beneficial effects achieved through these treatments [4].

## 3. Oxygen Homeostasis

Oxygen is essential for all aerobic organisms. Normoxia is defined as the normal oxygenation conditions necessary for the proper execution of physiological processes.

The oxygen level within the mitochondria [2] is very low, so even small variations act as potent triggers for metabolic signaling. Mitochondria play a central role in oxygen utilization, as cellular respiration occurs there. ATP, the cell’s energy source, is produced from glucose, with oxygen acting as the final electron acceptor in the respiratory chain. During this process, small amounts of reactive oxygen species (ROS) are generated, which are molecules capable of reacting with other molecules. 

**Figure 1:**
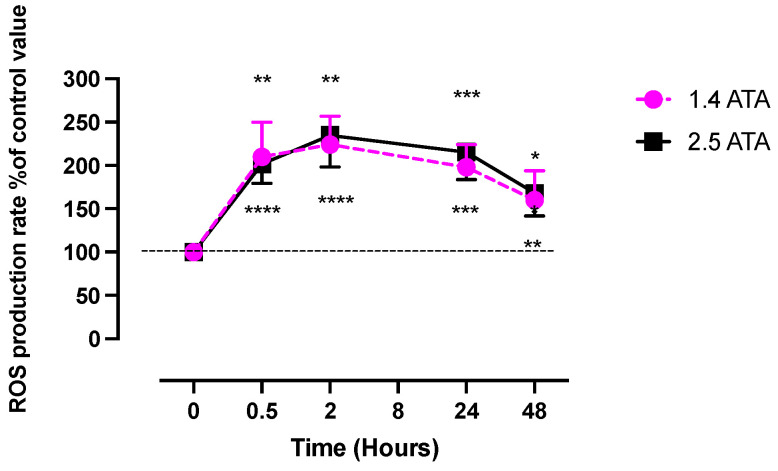
Percentual variations in ROS (plasma) production after 60 min of oxygen breathing of mild (1.4 ATA, n = 6) or high hyperbaric exposure (2.5 ATA, n = 8). Levels of oxygen are shown in the figure legend. Results are expressed as mean percentage change of control values (*: *p* < 0.05, **: *p* < 0.01, ***: *p* < 0.001, ****: *p* < 0.0001; RM-ANOVA and Dunnet’s post hoc test).

The hyperoxic state during hyperbaric oxygen therapy can elevate ROS production. Recently, it has been demonstrated that HBOT induces an increase in ROS with a kinetic profile similar to that of plasma reactive oxygen species at 1.4 ATA and 2.5 ATA. The peak production is attained around 2 h and persists above the baseline level for 48 h [5] (See Figure 1).

**Figure 2:**
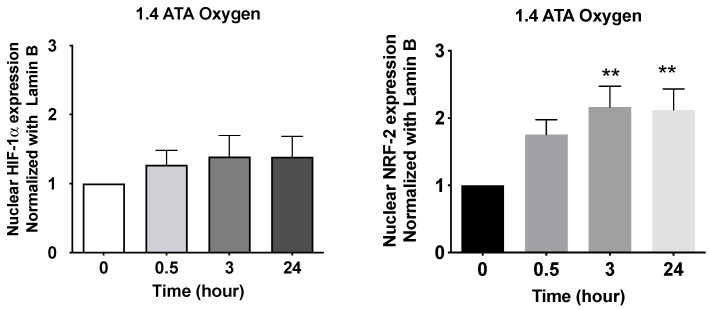
HIF-1α and NRF-2 nuclear translocation following 1 h of hyperbaric hyperoxia at 1.4 ATA hyperoxia in human subjects (N = 6) before and after the recovery to normoxic conditions. Results are expressed as fold change (mean *±* SEM) in comparison to baseline (0), which was set at 1. ** p < 0.01; for one-way ANOVA followed by Dunnett’s post hoc test (Modified from Fratantonio et al. (2021) [6].

The effects of ROS can be counterbalanced by the action of NRF2, a transcription factor that regulates the antioxidant response [3] (See Figure 2).

Its response magnitude and duration are also dependent on the oxygen dose applied as proposed by Fratantonio et al. (Fratantonio et al., 2021) [6].

When there is an excess of oxygen in the cell, and the endogenous antioxidant system is disrupted, oxidative stress is triggered. This can result in damage to lipids, proteins, and DNA. Furthermore, it is known that oxidative stress, inflammation, the immune system, and metabolism are interconnected. In this context, ROS is used as part of the signaling cascades of certain inflammatory cytokines, contributing to the development and/or progression of chronic inflammatory diseases [7].

On the contrary, in the case of persistent tissue hypoxia (oxygen deficiency), tissues can develop hypoxemic stress, which can lead to organ dysfunction and permanent functional impairment, as seen in conditions such as acute mountain sickness [8,9]. To prevent such damage, hypoxia serves as a key inducer of cellular gene expression, promoting various processes such as cellular protection and repair, angiogenesis, stem cell proliferation and differentiation, and more. Most of the induced gene expression is guided by transcription factors known as HIF-1 (hypoxia-inducible factors 1). Under normoxic conditions, HIF-1 levels are reduced since the reactive oxygen species (ROS) in the cell, products of cellular metabolism, promote their degradation. In a hypoxic situation, fewer ROS are produced, causing HIF-1 to stabilize, translocate to the nucleus, and promote the expression of approximately 200 genes essential for adapting to low-oxygen conditions. These genes include glycolytic enzymes for ATP synthesis, VEGF to induce angiogenesis, iNOS (inducible nitric oxide synthase), and other factors to enhance tissue oxygenation. HIF-1 is also associated with other processes such as mitochondrial biogenesis and aging through SIRT1 [2].

## 4. HBOT (Hyperbaric Oxygen Therapy)

In certain circumstances, hyperbaric oxygen therapy represents the primary treatment modality, while in others, it serves as a complement to surgical or pharmacological interventions [10].

Hyperbaric oxygen therapy is conducted using various types of chambers. Multiplace chambers are employed to simultaneously treat up to approximately 20 patients. These chambers are pressurized with compressed air, and 100% oxygen is administered via masks covering the nose and mouth or hoods that envelop the entire head. The atmosphere is carefully regulated and controlled concerning air and temperature. These chambers allow for the entry of equipment from intensive care units and the presence of medical personnel.

Monoplace Chambers are the most used type. These chambers can be pressurized with nearly 100% oxygen, and the patient directly breathes the oxygen from the chamber environment. They can also be pressurized with compressed air while the patient breathes oxygen (close to 100%) through masks, head hoods, or endotracheal tubes. This type of chamber offers individual patient management (isolation, useful for infections), making patients easy to observe. No special decompression procedures are required. More cost-effective than multiplace chambers. Requires less space than multiplace chambers. Fewer operators are needed. The design of monoplace chambers is ideal for caring for patients who do not require the presence of personnel within the chamber. Most bodily functions can be monitored externally, including the ventilator, which can be controlled from outside the chamber.

### 4.1. Fundamentals of Hyperoxic Treatment

The primary physiological effect of HBOT is the generation of hyperoxia, which allows for greater oxygen dilution in the blood plasma, and this process is independent of hemoglobin [11]. The oxygen supply chain begins in the lungs, where it is delivered through convection in the respiratory passages and blood vessels. It then diffuses through the capillary wall to the interstitium, with its ultimate destination being the mitochondria [2].

In the blood, oxygen is transported in two forms: a fraction bound to hemoglobin and a free fraction dissolved in the plasma. The amount of dissolved oxygen is proportional to the partial pressure of oxygen at a specific temperature, according to Henry’s Law [12]. Therefore, at a higher partial gas pressure, it tends to shift from a gaseous to a liquid state, with the reverse occurring when the pressure of the gas dissolved in the plasma decreases.

Furthermore, we should consider that the air we normally breathe is composed of 78% nitrogen, 21% oxygen, and the rest is made up of trace gases. According to Dalton’s Law, the total pressure exerted by a mixture of gases is equal to the sum of the partial pressures of the gases. This means that at sea level, 1 ATA (absolute pressure), the partial pressure of oxygen in the atmosphere is 159.6 mmHg. Exposure to pure oxygen at 1.5 ATA (absolute atmospheres) entails an oxygen pressure of 1140 mmHg. An increase in inspired oxygen pressure leads to an increase in alveolar oxygen pressure, which reaches 1053 mmHg when breathing 100% oxygen in a hyperbaric chamber at 1.5 ATA, compared to 102 mmHg when breathing air at 1 ATA, meaning it increases by a factor of 10.

At normal atmospheric pressures, only a limited portion of inspired oxygen is dissolved in the blood, but at higher pressures, as used in HBOT, it becomes possible to dissolve enough oxygen to meet the body’s usual needs. In line with this, Jain et al. described that arterial oxygen pressure increases with pressure, such that ideally, breathing 100% oxygen at 1.5 ATA results in a 10-fold increase in arterial oxygen compared to arterial oxygen under normal conditions (breathing air at 1 ATA), demonstrating the hyperoxia generated in HBOT at 1.5 ATA [13]. Ishihara et al. also reported an increase in dissolved oxygen using mid-pressure HBOT [14].

Breathing 100% oxygen in a high-pressure environment increases tissue oxygen tension. The diffusion of dissolved oxygen into tissues follows the mathematical model described by Krogh [15]. Using the Krogh model, the existence of radial and longitudinal pressure gradients (PpO_2_) is explained, based on the capillary radius and the arterial and venous endpoints, respectively. Combining these variables, the model allows the prediction of PpO_2_ in tissues. By administering O_2_ at close to 100% concentration in an environment at 1.45 atm, the oxygen penetration radius from capillaries to tissues is approximately ~75 µm, enabling the attainment of an arteriolar PpO_2_ of about 950 mmHg. This pressure is more than sufficient to ensure adequate oxygen supply to all tissues in the body through the diffusion and penetration of oxygen from plasma to all cells. Therefore, at 1.5 ATA, the minimum required PpO_2_ (20 mmHg) is achieved and considerably exceeded to cover the minimum average penetration radius needed to support cellular functions (~40 µm). Holbach et al. demonstrated that HBOT al 1.5 ATA increased arterial PO_2_ and cerebrospinal fluid oxygen pressure [16].

The increase in tissue oxygen levels after subjecting a patient to HBOT treatment has been widely demonstrated [17,18]. This increase persists even after the vasoconstriction generated by HBOT, as it does not impede the elevation of tissue partial oxygen pressure [18]. 

In addition, tissue hyperoxia has been demonstrated in the brains of rats exposed to hyperbaric therapy at 1.5 ATA [19]. These results were confirmed in clinical studies by Rockwold and colleagues, showing a significant increase in tissue oxygenation induced by HBOT at 1.5 ATA in the brains of patients [20]. These findings were also replicated in a phase II clinical trial, demonstrating hyperoxia generated at that pressure [21]. Moreover, the increase in tissue oxygenation achieved by HBOT at 1.5 ATA is capable of elevating ATP and NAD+ levels in brain tissue following a brain injury, meaning that hyperbaric oxygen supply can restore mitochondrial function after one hour of treatment. These effects were accompanied by a reduced loss of neuronal cells in the hippocampus, contributing to improved cognitive recovery [19]. 

Niza et al. observed that exposure to HBOT increases blood flow in peripheral tissue capillaries through parasympathetic activation induced by hyperoxia at 1.4 ATA [22].

It has been demonstrated that the increase in tissue oxygen induced by HBOT at 1.5 ATA using a monoplace chamber generates cellular responses, as described by Fratantonio et al. They reported that hyperoxia induced in healthy individuals at 1.5 ATA led to an increase in the expression of transcription factors that regulate cellular responses related to oxygen fluctuations. Additionally, an increase in glutathione and MMP9 (metalloproteinase 9) was observed, indicating that the treatment induces oxidative stress. These observations highlight a cellular response mediated by transcription factors induced by hyperoxia generated at 1.5 ATA [6].

Leveque et al. described the kinetics of ROS in healthy patients after 1 h of exposure to hyperbaric oxygen at 1.5 ATA and 2.5 ATA, up to 48 h following the treatment. The work demonstrated a significant increase in ROS due to hyperoxia generated at 1.5 ATA and the activation of antioxidant mechanisms. Both the ROS kinetics and the levels reached were comparable to the group that received HBOT at 2.5 ATA. Similarly, it was observed that the kinetics and levels of antioxidants were comparable at both pressures, while the immunomodulatory effects varied depending on the dosage [5].

The increase in ROS and antioxidants after HBOT was also described in the study by Bosco et al. They observed that these parameters remained elevated during the 20 daily sessions of HBOT conducted at the same pressures. At 1.5 ATA, ROS levels remained comparable between the two pressures until day 7 and returned to normal after completing the 20 sessions, while they continued to be elevated at 2.5 ATA. This was accompanied by an increase in antioxidant compounds with the same kinetics at both pressures, even up to one month after completing the treatment, indicating a shift in the redox balance toward the reduced state [23]. This allows us to conclude that at 1.5 ATA, there is a clear antioxidant effect that predominates over oxidative stress, which remains elevated one month after completing the 20 sessions tested. Furthermore, an anti-inflammatory effect was observed, evidenced by a decrease in IL-6 and miRNA expression, consistent with findings from other studies showing a decrease in proinflammatory cytokines and an increase in hemoglobin. These results demonstrate that intermittent hyperoxia generated at 1.5 ATA leads to an increase in ROS due to increased tissue oxygen, as well as cellular responses that contribute to an antioxidant, anti-inflammatory, and hemoglobin-increasing effect in a safer context without renal damage [24,25].

The effects of intermittent hyperoxia on processes such as inflammation, oxidative stress, and others can be explained through the phenomenon known as the “normobaric oxygen paradox” or “hypoxia-hyperoxia paradox” [2,26] depending on the pressure used. However, it is important to consider that both normobaric and hyperbaric intermittent exposures generate the same reactions. This paradox was proposed in 2006 when the authors observed that intermittent hyperoxia/normoxia exposure induced the synthesis of EPO (Erythropoietin). Essentially in a hyperoxic environment, there is more dissolved oxygen, leading to an increase in ROS. The adaptive response induces an increase in antioxidant proteins (scavengers) to counteract the increased ROS, preventing reactive species from causing damage to DNA and other cellular processes. When returning to normoxia, oxygen and ROS levels normalize, but the scavenger activity remains high for an extended period. When the hyperoxic exposure is repeated, the ROS/scavenger ratio is low because the half-life of scavengers is longer than that of ROS. This results in fewer ROS and, consequently, less degradation of HIF (Hypoxia-Inducible Factor) in the proteasome and more active HIF entering the nucleus to promote the transcription of EPO, VEGF, sirtuins, among others, leading to effects like hypoxia (angiogenesis, cell regeneration, etc.). In other words, the cell perceives intermittent hyperoxia as a state of hypoxia, promoting cellular processes induced by HIF but under normoxic conditions.

The effect of hyperbaric oxygen at 1.5 ATA on the immune system has been addressed by various authors who observed an increase in blood flow in capillaries [22]. As previously mentioned, U Nisa and colleagues recently demonstrated that IL-6 does not change, nor does IL-12, suggesting that the treatment would have an anti-inflammatory effect on healthy women exposed to HBOT at half-pressure. The authors also reported an increase in parasympathetic activity and an increase in natural killer cells (NKC), indicating an increase in the mobilization and proliferation of these cells involved in early immunity [27]. In other words, hyperoxia generated at 1.5 ATA in healthy women has an anti-inflammatory effect.

### 4.2. Physiological Effects of Hyperoxia Generated by HBOT

#### 4.2.1. Reversal of Hypoxia

Hyperbaric oxygen restores mitochondrial functionality and cellular respiration. Hyperoxia maintains the mitochondrial membrane potential and increases ATP production [28,29]. HBOT provides tissue perfusion exchange capability due to the increased instance of oxygen diffusion, distinguishing it from all other oxygen therapy methods. In this regard, HBOT treatments at 1.4–2.0 ATA for hypoxemia in critical COVID-19 patients continue to be a promising avenue [30]. Cannellotto et al. demonstrate that hyperbaric oxygen therapy at 1.45 atmospheres of pressure among patients with COVID-19 resulted in significantly faster resolution of hypoxia in those who received HBOT compared to control patients [31]. 

#### 4.2.2. Non-Hypoxemic Vasoconstriction

The increased oxygen available generated by HBOT induces vasoconstriction in small arteries and capillaries in healthy tissues, without impairing oxygenation, favoring the redistribution of blood flow to underperfused areas. The vasoconstriction produced is called “non-hypoxemic” because it does not counteract the effect of hyperoxia or deepen hypoxia in ischemic or poorly perfused tissues. Vasoconstriction, mediated by the central nervous system through α-adrenergic receptors, reduces blood flow by up to 20% without altering venous return, resulting in benefits for edema reduction at any level and a reduction in the inflammatory response. Additionally, HBOT can increase vascular tone, raising blood pressure and reducing heart rate [32,33].

#### 4.2.3. Angiogenesis

Hyperoxia stimulates neo-vascularization or the formation of new blood vessels through two processes: angiogenesis and vasculogenesis [1]. Numerous growth factors, transcription factors, hormones, and chemical mediators are involved in these mechanisms, including Hypoxia-Inducible Factor 1 (HIF-1), Vascular Endothelial Growth Factor (VEGF), Epidermal Growth Factor (EGF), Platelet-Derived Growth Factor (PDGF), interleukins (IL) [34]. Particularly, the pro-angiogenic effect triggered by HBOT is mediated by an increase in VEGF production, promoting the formation of new blood vessels after several sessions [35]. In bone marrow, HBOT affects the activity of medullary nitric oxide synthase (NOS) enzyme, which synthesizes nitric oxide (NO) and plays a role in the mobilization of stem cells, promoting neovascularization and healing [36].

#### 4.2.4. Proliferation and Stimulation of Stem Cells

HBOT generates the hyperoxia necessary for oxidative stress via nitric oxide synthase to release pluripotent stem cells from the bone marrow stroma [37]. The mobilization of progenitor stem cells (SPCs) due to has been demonstrated in healthy individuals and in patients receiving treatment for radionecrosis. Hyperbaric oxygen mobilizes endothelial progenitor cells (EPCs), which have been associated with endothelial cell generation and demonstrated vasculogenesis [38]. It also promotes the differentiation of neural stem cells into neurons and oligodendrocytes, and reduces the number of astrocytes in vitro, possibly by regulating the Wnt3 protein signaling pathway, beta-catenin, and bone morphogenetic protein 2 (BMP2). An up-regulation of neural cell proliferation is also observed within neurogenic niches in the adult brain [39]. 

#### 4.2.5. Collagen Synthesis

HBOT has been shown to increase the expression of type I pro-collagen genes in tendon and ligament healing and inhibits the expression of metalloproteinases [40]. Hyperoxia induced by HBOT activates the expression of growth factors for fibroblast activation, resulting in a significant increase in type I and type III collagen synthesis related to local nitric oxide production at the wound site during healing. Oxygen is not only important in collagen maturation, necessary for stable collagen formation, but it also increases collagen precursor synthesis, favoring the synthesis and repair of various types of tissues [41].

#### 4.2.6. Osteogenesis

Hyperbaric oxygenation increases oxygen perfusion even in inflamed or infected bone, promoting angiogenesis that nourishes bone tissue and subsequently osteogenesis. Hyperoxia also promotes bone tissue differentiation. In this regard, it has been demonstrated that HBOT at 1.5 ATA and 2.4 ATA HBO treatments stimulates early osteogenic differentiation increasing the number of osteoblasts, calcium deposition, alkaline phosphatase activity and bone nodule [42]. Osteogenesis is accelerated compared to osteoblasts that did not receive hyperbaric oxygen [43]. Increased oxidative stress triggers the bone resorption process associated with aging and osteoporosis [44]. 

#### 4.2.7. Antimicrobial Effect

Hyperoxia generated by HBOT reverses tissue hypoxia, which is a conducive environment for the development and proliferation of anaerobic and microaerophilic bacteria. It enhances the bactericidal effectiveness of antimicrobials against biofilm-producing bacteria, such as *Pseudomonas aeruginosa*, demonstrating a synergistic bactericidal action with antimicrobials in biofilm-producing bacteria [45]. It increases the concentration of reactive oxygen species (which have intrinsic bactericidal activity against bacteria), promotes innate and adaptive immune responses, enhances the phagocytosis of bacteria like *Staphylococcus aureus*, supports mitochondrial function, reverses mitochondrial damage, and provides oxygen for metabolism and reactive oxygen species production. This provides the necessary elements to promote cellular immunity and increase defenses against various pathogens. It inhibits the action of bacterial toxins that are important in infectious diseases [46].

#### 4.2.8. Neural Function and Neuroprotection

Hyperoxia generated by HBOT increases Hypoxia-Inducible Factor 1 alpha (HIF-1alpha) and the subsequent synthesis of the cytokine erythropoietin (EPO), resulting in significant neuroprotective effects [47,48]. Hyperbaric oxygen, as a preconditioning method, also confers hypoxia tolerance by providing neuroprotection in the cerebral cortex, hippocampus, and spinal cord. It promotes the inhibition of neuronal apoptosis, reduces Caspase-3 expression, preventing DNA fragmentation and preserving cellular integrity [49].

#### 4.2.9. Mitochondrial Function, Oxidative Stress, and Inflammatory Response

HBOT induces the production of reactive oxygen species (ROS), which can act as signals measuring physiological responses in mitochondria. Hyperbaric oxygen preserves mitochondrial integrity by maintaining the mitochondrial membrane potential and reducing the mitochondrial apoptosis pathway. It restores cellular respiration in cases of poisoning [50]. Additionally, hyperbaric oxygen treatment induces mitophagy [51]. Mitophagy activation is associated with a significant decrease in neuropathic pain [52]. Increased dissolved oxygen leads to a significant reduction in neuropathic pain. Hyperoxia can simultaneously stimulate an increase in ROS levels and antioxidant species, both enzymatic and non-enzymatic [53]. It can induce protection against oxidative stimuli in endothelial cells by overexpressing antioxidant genes following multiple hyperbaric oxygenation sessions in some tissues [23]. The short-term effect of HBOT generates stress and causes mitochondria to decrease their activity, partially reducing ROS production. However, in the long term, antioxidant activity increases, helping mitochondria function without altering the redox balance and even improving their activity [54]. The anti-inflammatory action mediated by HBOT leads to vasoconstriction, reducing edema and the inflammatory response. It also reduces the production and release of pro-inflammatory cytokines by neutrophils and monocytes [1,55]. HBOT has effects on cytokine production, reducing interleukin 1 (IL-1), interleukin 6 (IL-6), and tumor necrosis factor alpha (TNF-alpha), which are pro-inflammatory. VEGF (Vascular Endothelial Growth Factor), TGF-beta1 (Transforming Growth Factor Beta 1), and PDGF-beta (Platelet-Derived Growth Factor) exhibit biphasic release patterns. This means that they are released under both hypoxic and hyperoxic conditions. However, hyperoxia-stimulated release is greater, facilitating the healing process [56].

#### 4.2.10. Regeneration

Studies have reported that hyperoxia generated by HBOT promotes regeneration mechanisms in various tissues, including the brain, spinal cord, bone, cartilage, cardiovascular tissue, and muscle. The observed regeneration can be explained by the effects of HBOT on the following processes: apoptosis, stem cell mobilization, oxidative stress response, inflammation, tissue remodeling, angiogenesis, cell–cell adhesion mechanisms, tissue regeneration, differentiation, and proliferation [57]. HBOT promotes axon regeneration, as hyperoxia-induced accumulation of anti-inflammatory cytokines and macrophage accumulation.

## 5. Safety

Hyperbaric oxygen therapy is an important complementary treatment for various pathologies, as mentioned earlier, and is currently a safe and available therapy with some reported adverse effects primarily at high pressures. The incidence of adverse events associated with HBOT is rare and generally not severe, encompassing conditions such as otic/sinus barotrauma, confinement anxiety, hypoglycemia, oxygen toxicity, ocular side effects, pneumothorax, seizure, and shortness of breath [58]. Absolute contraindications have been defined, such as untreated pneumothorax, and relative contraindications include the use of certain medications, asthma, pregnancy, claustrophobia, Chronic Obstructive Pulmonary Disease (COPD), insulin-dependent diabetes mellitus, or acute hypoglycemia, among others [59]. It is imperative to systematically assess the risk and benefit profiles at various treatment pressures and durations to standardize treatment protocols and enhance patient care.

Monge et al. demonstrated that HBOT at 1.45 atm using a portable chamber increases blood oxygen saturation, demonstrating hyperoxia, and was safer than other reported chambers, as adverse effects were milder, and no barotrauma was reported [60]. Similarly, Jaim and colleagues did not report adverse effects [13].

Other studies have shown the safety of treatment at 1.5 atm. Ilni and colleagues reported in healthy volunteers that at half-pressure, there were no observed changes in lung capacity [61]. Furthermore, there was no increase in IL6 and IL-8 in bronchoalveolar lavage, and no pulmonary or cerebral oxygen toxicity was observed [62]. Laboratory parameters studied in healthy individuals receiving HBOT at 1.45 atm showed no changes in hematological or hemostatic parameters, as well as in markers of inflammation and acute phase [63].

## 6. Methods

A literature search was performed in Pubmed to identify studies, conducted in the las 20 years that explore the use of HBOT at pressure above 1.45–1.5 ATA. The following search string was used: (“hyperbaric oxygenation” AND “hyperoxia” AND; “mild pressure”) OR (“Hyperbaric Oxygen Therapy effects” AND “oxidative stress and HBOT” AND “1.5 ATA”). The review was focus on clinical approach in adult healthy subject or subjects with any pathology to whom tissue oxygen levels were measured. The search was restricted to articles published in English in peer-reviewed journals. No restriction on study design was imposed. Abstract presentations and conference proceedings were excluded.

Studies were manually selected based on the title and abstract. (See Table 1). Selected studies were read thoroughly to identify those suitable for inclusion in this review. We extracted the demographic and experimental data from the selected studies. For each study, the following relevant information was extracted and summarized: characteristics of the investigated population; oxygen administration protocols the experimental and/or clinical settings of application; and the main results of the studies in terms of level of oxygen and/or level of ROS enhancing effect on HIF-1α pathway.

## 7. Conclusions

Hyperbaric oxygen treatment at 1.5 ATA increases dissolved oxygen levels, generating tissue hyperoxia and counteracting the effects of hypoxia by supplying the necessary oxygen to restore cellular respiration and mitochondrial function. The effect of HBOT also involves cellular responses that counteract oxidative stress effects by activating antioxidant mechanisms, and it acts on multiple processes such as angiogenesis and inflammation, among others, producing beneficial effects on various pathologies. These beneficial effects have been reported in a wide range of protocols, including various pressures, exposure times, and session numbers. Therefore, it will be necessary to conduct rigorous studies to adjust these variables for HBO therapy, whether as an independent or adjuvant treatment, in the numerous pathologies in which it may yield benefits, potentially leading to transformative therapeutic advancements in the future.

## Figures and Tables

**Table 1:** A summary of the main human studies demonstrating hyperoxia with hyperbaric oxygen treatment at mild pressure.

Author	Type of Study	Sample Size	Intervention	Main Results
Bosco et al. (2021) [23]	Randomized, patient-blinded, controlled trial (NCT04366427)	22 healthy humans.	100% O_2_, HBOT 1.5 ATA, 60 min. 20 treatments	In plasmatic samples: Increase in ROS levels until day 14, followed by a decrease at the end of the treatment. Increase in total antioxidant capacity. Increase in glutathione and reduced cysteine. Decrease in IL-6 and IL-10.
Leveque et al. (2023) [5]	Human experimental study	47 healthy non-smoking Caucasian subjects	100% O_2_, HBOT 1.4 ATA, 60 min. 1 treatment.	Increase of plasmatic ROS production similar kinetic respect 2.5 ATA. Increase in cysteinylglycine. Plasmatic Nitric oxide metabolites decrease after 2 h and return normal level al 24 h
Fratantorio et al. (2021) [6]	Human experimental study	20 healthy humans	1 h exposure to normobaric oxygen FiO_2_ 0.3 vs. normobaric oxygen FiO_2_ 1.0 vs. hyperbaric oxygen 1.4 bar FiO_2_ 1.4	Increase in NRF2 nuclear level and not significative changes in HIF after the first session in peripheral blood mononuclear cells. Increase glutathione and matrix metalloproteinase (MMPs) in plasma.
Niza et al. (2023) [27]	Clinical Trial (crossover randomized experimental study)	16 healthy women	1.4 ATA, with 35.0–39.5% oxygen concentration and 18 L of oxygen per minute injected into the hyperbaric chamber for 70 min.	Parasympathetic activity (Electrocardiography Analysis) was significantly increased. NK cells were increased without changes IL-6 and IL-12p70 protein levels in blood samples.
Rockswold et al. (2010) [20]	Randomized clinical trial	24 patients	100% FiO_2_ (fraction of inspired oxygen) delivered for 60 min at 1.5 ATA. The NBH treatment consisted of 100% FiO_2_ given for 3 h at 1.0 ATA	Mean brain tissue PO_2_ levels were significantly increased after HBO_2_ therapy.
Rockswold et al. (2013) [21]	Prospective randomized Phase II clinical trial (NCT00170352)	45 patients treated for severe TBI	100% FiO_2_ delivered for 60 min at 1.5 ATA followed by 3 h at 1.0 ATA	Brain tissue PO_2_ in both the noninjured and pericontusional brain rose during the treatment sessions to approximately 600% of the control group value. Decrease of microdialysate lactate in the pericontusional brain without change in the noninjured brain.

## Data Availability

Not applicable.
